# Early Detection of Monopolar Radiofrequency Tip Rupture Using Polarized Dermoscopy: A Preventive Approach Against Treatment‐Related Burns

**DOI:** 10.1111/jocd.70678

**Published:** 2026-01-18

**Authors:** Shiou‐Han Wang, Patricia Yu‐Chun Peng, Hsien‐Li Peter Peng

**Affiliations:** ^1^ Department of Dermatology National Taiwan University Hospital Taipei Taiwan; ^2^ Kaohsiung Medical University Hospital Kaohsiung Taiwan; ^3^ P‐Skin Professional Clinic Kaohsiung Taiwan; ^4^ Department of Dermatology, Tri‐Service General Hospital National Defense Medical Center Taipei Taiwan

**Keywords:** adverse event prevention, capacitive membrane rupture, dermoscopy, monopolar radiofrequency, thermoelectric burn

## Abstract

**Background:**

Monopolar radiofrequency (MRF) devices use a capacitive membrane (CM) tip to deliver uniform heat to tissue. A peri‐procedural CM rupture, while rare, risks causing thermoelectric burns.

**Aims:**

To evaluate whether using polarized dermoscopy and preventive measures can detect CM ruptures early and prevent burns in MRF treatments.

**Methods:**

We retrospectively reviewed all patients who underwent monopolar radiofrequency (MRF) treatment at a single dermatologic clinic between 2012 and 2024 and experienced a peri‐procedural capacitive membrane (CM) rupture event. Non‐contact polarized‐dermoscopy‐aided CM rupture detection and relevant preventive measures (cryogen checking, shaving, and patient education) were introduced since 2017. Clinical photographs were assessed by two board‐certified dermatologists to verify thermoelectric burns.

**Results:**

Among 13 cases of capacitive membrane (CM) rupture identified between 2012 and 2024, 8 ruptures were detected by naked‐eye observation between 2012 and 2016, and all patients (8/8, 100%) developed thermoelectric burns. From 2017 onward, 5 ruptures were detected using non‐contact polarized dermoscopy, and none of the associated patients (0/5, 0%) developed burns. This difference was statistically significant (*p* = 0.0008). In addition, the implementation of preventive measures—such as cryogen inspection and shaving protocols—was associated with a reduced number of CM rupture events (*p* = 0.0063).

**Conclusions:**

Polarized dermoscopy, combined with preventive steps, enabled early detection of CM tip ruptures and prevented MRF‐induced burns.

## 1 Introduction

Noninvasive monopolar radiofrequency (MRF) technology is increasingly used in aesthetic medicine to achieve skin tightening and neocollagenesis through thermoelectric effects [1]. MRF functions by delivering sustained heat to the targeted tissue at a suitable depth (usually 3–6 mm); it does so by using a disposable capacitive membrane (CM) tip to create a uniform local electrical field, ensuring even energy delivery. Unfortunately, the unpredictable rupture of the CM poses a significant risk for thermoelectric burns due to an uneven distribution of heat [2]. In one commonly‐used MRF system (Thermage CPT, Solta Medical Inc., Bothell, USA), which utilizes a polyimide‐based capacitive membrane (CM), the manufacturer's Technical Bulletin reports the probability of CM rupture to be < 1% [3]. Based on the 2018 data from Taiwan's distributor, an estimated CM rupture incidence was calculated to be 0.15%–0.2% through dividing the total shipment volume by the number of tips recalled due to ruptures. Given that CM ruptures are not always visible to the naked eye, and prolonged use of CM tips after rupture can cause burns and other suboptimal therapeutic effects, a monitoring and early detection method to evaluate CM integrity and find ruptures as early as possible can be of great benefit.

The physical properties of polarized light—its ability to significantly reduce surface reflections and penetrate 60–100 μm into the skin without interface fluids—make it a suitable tool for visualizing subsurface structures. Polarized dermoscopy, by utilizing cross‐polarized illumination, allows for non‐contact, high‐contrast visualization of subtle surface irregularities [4, 5]. This makes it a promising method for detecting CM ruptures during monopolar RF procedures, which are often not apparent to the naked eye.

## 2 Methods

We conducted a retrospective analysis of all patients who received MRF treatment at a single dermatologic clinic between 2012 and 2024. Patients were included if their treatment was associated with a peri‐procedural CM rupture event.

Beginning in 2017, the clinic implemented a standardized bundle of preventive measures to reduce rupture risk and enable early detection. These included non‐contact polarized dermoscopy for CM inspection, cryogen spray verification prior to treatment, shaving of facial hair for male patients, and real‐time patient education regarding abnormal sensations.

Clinical outcomes were evaluated based on the presence or absence of thermoelectric burns, as confirmed by pre‐ and post‐treatment photographs, follow‐up documentation, and visual inspection of the ruptured handpiece tip. Two board‐certified dermatologists independently reviewed all photographs to confirm the diagnosis of CM rupture and treatment‐related burns.

Statistical analysis was performed using Fisher's exact test to assess the association between polarized dermoscopy use, implementation of preventive measures, and the incidence of burns.

## 3 Results

**FIGURE 1:**
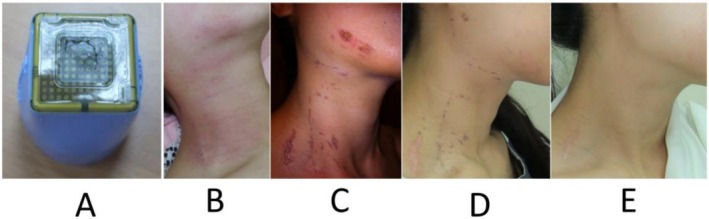
An example of a prominent peri‐procedural capacitive membrane (CM) rupture and the resulting severe thermoelectric burns. (A) Ruptured CM on handpiece. (B) The patient treated with the ruptured CM handpiece immediately after treatment. (C) 4 days after treatment, showing prominent degree I thermoelectric burns. (D) 8 days after treatment. (E) 14 days after treatment.

Between 2012 and 2016, eight cases of CM rupture were reported. All were identified solely by visual inspection of the treatment tip, and all patients (8/8, 100%) subsequently developed visible thermoelectric burns, as confirmed independently by two board‐certified dermatologists (Figure 1). Notably, one male patient who did not undergo shaving prior to treatment (part of the standardized preventive bundle) experienced linear burns along areas of facial hair, suggesting mechanical abrasion as a possible contributing factor.

**FIGURE 2:**
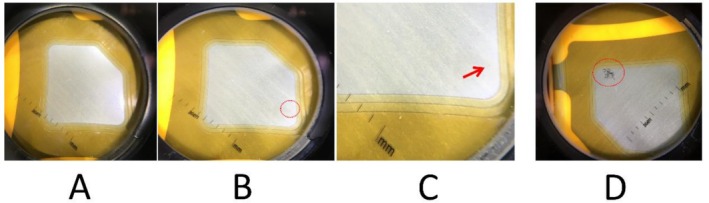
Very early detection of a miniscule capacitive membrane rupture with non‐contact polarized dermoscopy. (A) The rupture is invisible to non‐polarized dermoscopy, but (B and C) visible to polarized dermoscopy. (D) Another case demonstrated that, without early detection using a dermoscope, the ruptures could become significant enough to cause severe thermoelectrical burns.

From 2017 to 2024, five cases of CM rupture were detected using non‐contact polarized dermoscopy following the implementation of preventive protocols. None of the patients in this group (0/5, 0%) developed burns (Tables 1 and 2). In all five dermoscopy‐detected cases, polarized dermoscopy successfully revealed structural disruption of the CM, whereas non‐polarized dermoscopy failed to visualize the rupture in each corresponding case (Figure 2).

**TABLE 1:** Summary of preventive measures versus total number of capacitive membrane ruptures in 2012–2024.

Period	Total CM ruptures	Preventive measures	*p*
2012–16	8	No	0.0063
2017–24	5	Yes	

*Note:* Preventive measures include pre‐treatment shaving (for male patients), cryogen double‐checking, and patient education; *p*‐value was calculated using Fisher's exact test.

Abbreviation: CM, capacitive membrane.

**TABLE 2:** Summary of rupture detection method versus rate of burns in 2012–2024.

Period	Total CM ruptures	Detection method	Burn cases	Burn rate (%)	*p*
2012–16	8	Naked eye	8	100	0.0008
2017–24	5	Polarized dermoscopy	0	0	

*Note:* *p*‐value was calculated using Fisher's exact test.

Abbreviation: CM, capacitive membrane.

### 3.1 Statistical Analysis

Fisher's exact test revealed a statistically significant association between the use of polarized dermoscopy and the absence of treatment‐related burns (*p* = 0.0008), supporting its role in early rupture detection and burn prevention. In addition, implementation of the bundled preventive measures was associated with a significant reduction in the overall incidence of CM rupture events (*p* = 0.0063) (Tables 1 and 2).

## 4 Discussion

### 4.1 Clinical Relevance of CM Rupture in MRF

Although MRF treatments are generally considered safe and are widely used in aesthetic medicine, dermal burns have occasionally been reported [2]. CM rupture is an extremely rare complication, but precisely because of its rarity, many users may be unfamiliar with its presentation and management. Therefore, raising awareness and providing effective early detection strategies for such events is essential to maintaining procedural safety. According to the manufacturer's Technical Bulletin [3], it is recommended to perform a visual inspection of the CM at the beginning of the procedure to check for any signs of damage or accumulation of foreign matter, and perform the membrane inspection again after every 50 pulses to ensure the integrity of the membrane. However, this approach could be impractical due to the low probability of peri‐procedural CM rupture. In clinical practice, this can impede treatment efficiency and may be tedious to implement, and even with such measures, a burn may already have occurred by the time the rupture is detectable by the naked eye.

### 4.2 Material Considerations: Polyimide Versus PET

The material composition of the capacitive membrane (CM) plays a critical role in rupture resistance, with polyimide offering superior thermal and mechanical durability compared to polyethylene terephthalate (PET) [6, 7]. Polyimide films are widely used in RF device tips due to their excellent thermal stability and chemical inertness. However, recent reviews suggest that their structural integrity may degrade over time under physiological conditions due to water absorption, thermal stress, or manufacturing variability [8]. These factors may contribute to membrane micro‐failures during repeated or prolonged energy delivery.

Although polyimide is more resistant to damage than PET—owing to its aromatic heterocyclic structure and stronger intermolecular forces [8, 9, 10]—14 polyimide CM rupture events still occurred in our single‐clinic based study over 12 years, demonstrating a low but not insignificant risk. Several aesthetic MRF devices currently use PET‐based membranes, which may be even more susceptible to structural compromise. Therefore, early detection and prompt response to CM ruptures are critical, especially for systems utilizing less durable materials.

### 4.3 Before Treatment: Preventive Measures Bundle to Minimize Risk

CM rupture may result from several interacting factors, including thermal accumulation, mechanical abrasion, and material fatigue. In our analysis, the three most commonly implicated mechanisms were: (1) insufficient cryogen delivery, leading to inadequate tip cooling; (2) friction from facial hair causing surface abrasion; and (3) degradation of membrane material over repeated use. While the first two causes can be actively prevented, material fatigue may only be mitigated through early detection.

To address these risks, we implemented the following preventive strategies:

#### 4.3.1 Ensuring the Presence of Sufficient Cryogen

**FIGURE 3:**
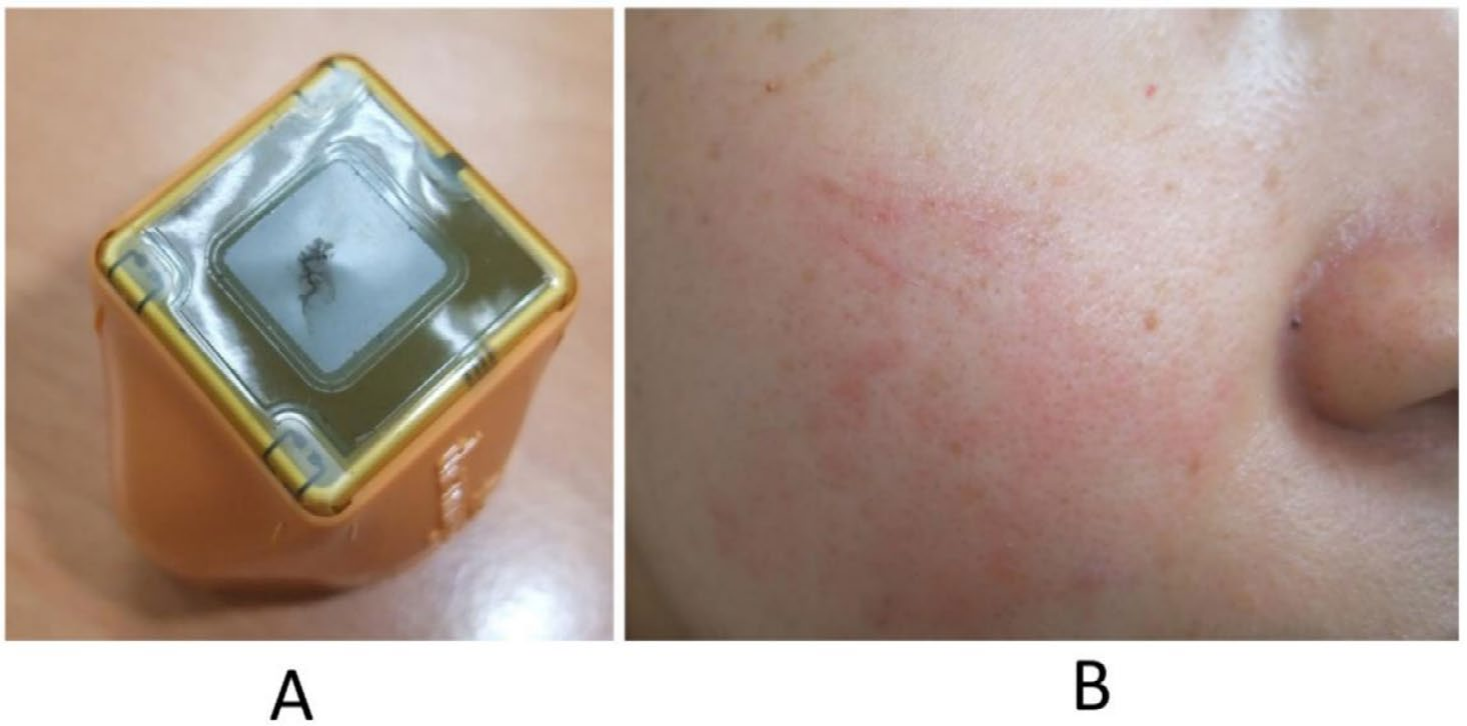
Peri‐procedural capacitive membrane (CM) rupture associated with insufficient cryogen. In this case, the radiofrequency pulses were triggered immediately after changing the handpiece without adequate cryogen in the lumen. (A) Ruptured CM on handpiece. (B) The patient treated with the ruptured CM handpiece showed degree I thermoelectric burns immediately after treatment.

Before replacing the handle or delivering multiple pulses, the physician should ensure that sufficient cryogen is present. The protective role of cryogen in preventing thermoelectric burns cannot be overstated [11], but cryogen also serves as a protectant for the CM. For most MRF devices on the market, the CM is typically made from polyimide or PET [6, 7]. While this material possesses heat resistance and insulating properties, it remains vulnerable to damage in rare instances if the heat distribution is too asymmetrical or localized (i.e., when not adequately protected by cryogen). This can lead to the formation of tiny pinhole‐like perforations, which may then expand into dendritic cracks (Figure 3).

#### 4.3.2 Ensuring That Male Patients Shave Before Treatment

**FIGURE 4:**
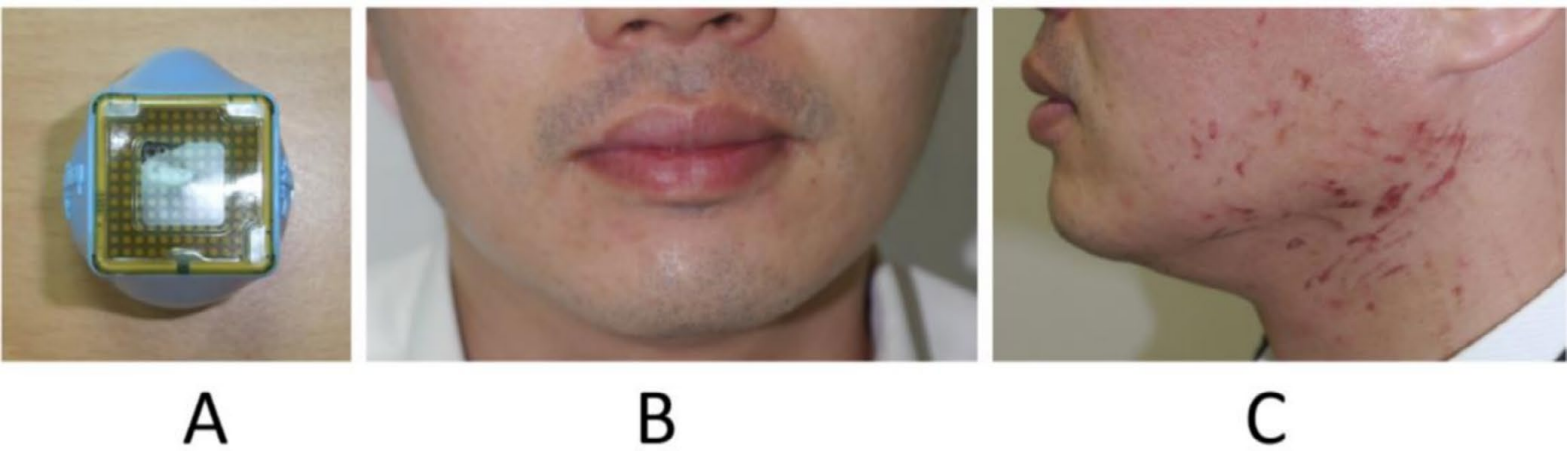
Peri‐procedural CM rupture, suspected to be associated with beard‐related abrasion. (A) Ruptured CM on handpiece. (B) The patient treated with the ruptured CM handpiece, before treatment; note the unshaved beard with prominent stubble. (C) Day 3 post‐treatment, showing prominent linear degree I thermoelectric burns over the bearded areas.

Shaving reduces mechanical contact between facial hair and the membrane surface, which may otherwise cause abrasion during repeated contact [2]. As illustrated in Figure 4, one unshaved male patient developed severe linear burns associated with peri‐procedural CM rupture, suspected to be caused by beard‐related abrasion.

#### 4.3.3 Optimizing Patient and Physician Education and Feedback

Educating both patients and physicians to recognize early warning signs—such as electrical sensations, localized heat, or burning odors—can greatly improve the early detection of CM rupture. These clinical cues are essential for prompting timely inspection using non‐contact polarized dermoscopy. To ensure accurate feedback, patients should not be sedated during MRF procedures, as sedation may obscure sensory awareness and increase the risk of undetected thermoelectric burns.

### 4.4 During Treatment: Polarized Dermoscopy as Effective Detection

Effective prevention of CM rupture and associated burns depends on timely recognition of warning signs during treatment and the implementation of targeted preventive strategies. Clinical warning signs—such as abnormal current sensation or a burning odor during treatment—should prompt immediate inspection of the capacitive membrane (CM) using polarized dermoscopy.

Specifically, we recommend CM dermoscopy under the following conditions:

1. Abnormal current sensation reported by the patient—this may indicate compromised insulation or membrane rupture and warrants immediate evaluation.
2. A faint burning odor, particularly one resembling electrocautery, should raise suspicion of a membrane tear causing localized overheating of tissue or device elements.

### 4.5 Implications of Preventive Measures With Polarized Dermoscopy

**FIGURE 5:**
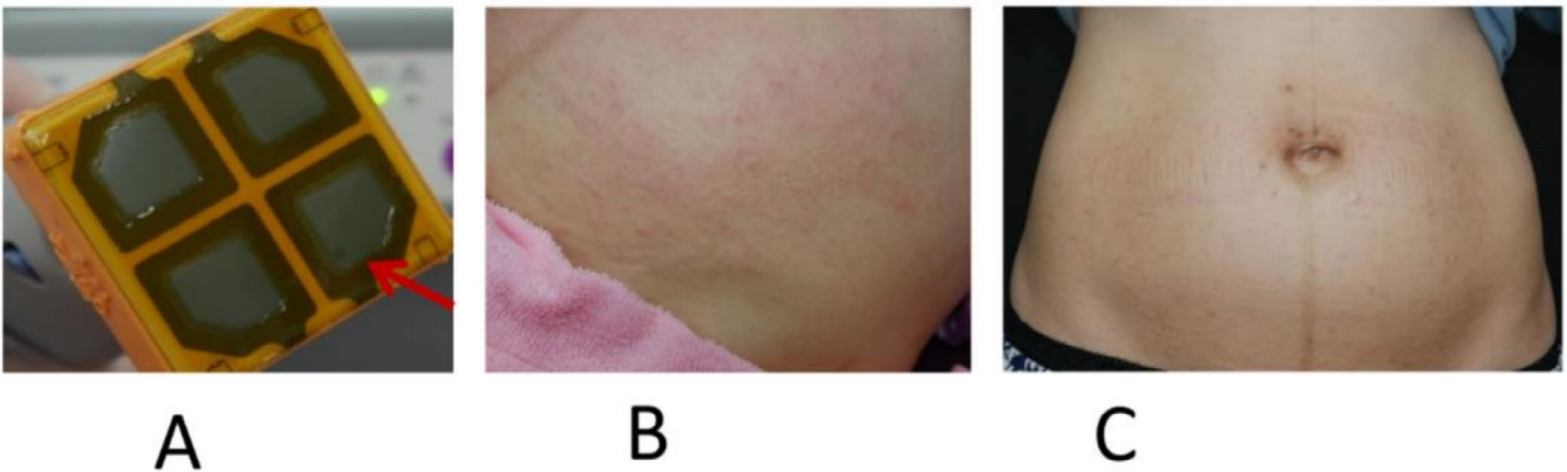
Peri‐procedural capacitive membrane (CM) rupture, detected early using non‐contact polarized dermoscopy. (A) Ruptured CM on handpiece. (B) The patient treated with the ruptured CM handpiece showed mild erythema immediately after treatment, raising concerns of thermoelectric burn. (C) The erythema subsided less than a day after treatment.

Our findings indicate that the implementation of polarized dermoscopy may enable timely identification of CM rupture and might help prevent thermoelectric burns. These findings provide preliminary support for the use of non‐contact polarized dermoscopy to enhance safety in MRF treatments. Figure 5 shows an example of a peri‐procedural CM rupture detected via polarized dermoscopy, resulting in only transient erythema. Furthermore, bundled preventive measures, including cryogen double‐checking, pre‐treatment shaving, and patient education, were strongly associated with a reduced burn rate even in the event of CM rupture. In the interest of patient safety, we did not devise a negative control group in which CM ruptures were checked using polarized dermoscopy but preventive measures were not used; therefore, the two interventions could not be separately analyzed, but the absence of any burns after 2017 indicates that preventive measures work well in combination with polarized dermoscopy‐assisted CM rupture detection to reduce overall complications.

### 4.6 Practical Considerations and Learning Curve for Dermoscopy Integration

In real‐world practice, integrating non‐contact polarized dermoscopy into MRF workflows inevitably introduces additional equipment and workflow considerations. A handheld polarized dermoscope represents a modest upfront investment for most dermatologic clinics, and operators need a short learning period to become familiar with recognizing early CM disruption patterns. In our single‐center experience, this learning curve was limited and did not materially prolong overall treatment time. Nonetheless, clinicians should be aware that each dermoscopic inspection adds a brief pause to the procedure, and practice settings with high patient throughput may need to adapt their scheduling and staff training accordingly.

### 4.7 Limitations

CM rupture is an extremely rare complication of MRF, with an estimated incidence of < 0.2%. This study is based on a single‐center, retrospective analysis spanning 12 years, which may limit the generalizability of the findings. Rather than causing undue concern, the purpose of this report is to raise awareness and improve preparedness for rare but potentially preventable adverse events, thereby contributing to safer clinical practice. Furthermore, since this retrospective analysis focused on CM rupture events, the exact total number of MRF procedures performed during the study period was not systematically captured. As a result, we were unable to calculate a precise incidence rate of CM rupture and instead compared the frequency of rupture events before and after implementation of the preventive bundle.

## 5 Conclusion

This study highlights the critical importance of CM integrity monitoring during MRF treatment and offers non‐contact polarized dermoscopy as a viable method for early detection of ruptured CM. This can be augmented by several preventive measures, including cryogen double‐checking, ensuring that male patients shave, and implementing patient education and feedback. Some limitations include the retrospective, single‐center nature of the study, the small sample size, and the lack of a systematically recorded denominator for all MRF procedures during the study period. Consequently, we could not calculate a formal incidence rate of CM rupture, and our findings are restricted to the observed frequency of rupture events in this single clinic. While more studies are required before our findings can be definitively recommended, our data support non‐contact polarized dermoscopy as a practical adjunct for early CM rupture detection in routine MRF practice. Future multicenter studies are warranted to validate these observations across different device platforms and patient populations, and collaboration with device manufacturers may allow integration of dermoscopy‐based or automated membrane integrity checks into next‐generation MRF systems. Such efforts could further reduce the already low risk of thermoelectric burns and help ensure safer, more predictable outcomes for both patients and physicians.

## Author Contributions

S.‐H.W. was responsible for the conception, data collection, analysis, and writing of this manuscript. P.Y.‐C.P. contributed to case review, verification of dermoscopic findings, textual editing, and validation of statistical logic. H.‐L.P.P. was responsible for data and analytical method verification, manuscript writing, and revision. All authors reviewed and approved the final version of the manuscript.

## Ethics Statement

The authors have nothing to report.

## Consent

Informed consent has been obtained from all patients to have their photograph published.

## Conflicts of Interest

S.H.W. previously served as a consultant for Solta Medical Inc., the manufacturer of the Thermage device. This role was unrelated to the current study, and no financial or material support was provided for this work. P.Y.‐C.P. and H.‐L.P.P. declare no conflicts of interest.

## Data Availability

The data that support the findings of this study are available from the corresponding author upon reasonable request.

## References

[1] A. Chapas , B. S. Biesman , H. H. L. Chan , et al., “Consensus Recommendations for 4th Generation Non‐Microneedling Monopolar Radiofrequency for Skin Tightening: A Delphi Consensus Panel,” Journal of Drugs in Dermatology 19, no. 1 (2020): 20–26, 10.36849/JDD.2020.4807. 31985194

[2] F. A. Mayoral and J. M. Vega , “Multiple Facial Burns With the New Thermage CPT System,” Journal of Drugs in Dermatology 10, no. 11 (2011): 1320–1321. 22052316

[3] Solta Medical, Inc ., “Treatment Tip Membrane Failure During Thermage CPT Treatment—Risk of Patient Injury,” Technical Bulletin No. TB‐19, Rev. A, 2010.

[4] B. Nirmal , “Dermatoscopy: Physics and Principles,” Indian Journal of Dermatopathology and Diagnostic Dermatology 4, no. 2 (2017): 27, 10.4103/ijdpdd.ijdpdd_13_17.

[5] Y. Pan , D. S. Gareau , A. Scope , M. Rajadhyaksha , N. A. Mullani , and A. A. Marghoob , “Polarized and Nonpolarized Dermoscopy: The Explanation for the Observed Differences,” Archives of Dermatology 144, no. 6 (2008): 828–829, 10.1001/archderm.144.6.828. 18559791

[6] E. W. Knowlton and Thermage, Inc ., “Method for Treating Skin and Underlying Tissue,” U.S. Patent No. 7,189,230 B2, 2007.

[7] WON TECH, Co., Ltd ., “Radio Frequency Treatment System,” U.S. FDA Premarket Notification, 510(k) No. K221989, 2022.

[8] C. P. Constantin , M. Aflori , R. F. Damian , and R. D. Rusu , “Biocompatibility of Polyimides: A Mini‐Review,” Materials (Basel) 12, no. 19 (2019): 3166, 10.3390/ma12193166. 31569679 PMC6804260

[9] L. Turnbull , J. J. Liggat , and W. A. MacDonald , “Thermal Degradation Chemistry of Poly(Ethylene Naphthalate) – A Study by Thermal Volatilisation Analysis,” Polymer Degradation and Stability 98, no. 11 (2013): 2244–2258, 10.1016/j.polymdegradstab.2013.08.018.

[10] D. R. D'hooge , M. Edeleva , C. Fiorillo , et al., “Molecular and Material Property Variations During the Ideal Degradation and Mechanical Recycling of PET,” RSC Sustainability 2 (2024): 3596–3637, 10.1039/d4su00485j.

[11] R. A. Weiss , M. A. Weiss , G. Munavalli , and K. L. Beasley , “Monopolar Radiofrequency Facial Tightening: A Retrospective Analysis of Efficacy and Safety in Over 600 Treatments,” Journal of Drugs in Dermatology 5, no. 8 (2006): 707–712. 16989184

